# EHMTI-0322. Effect of cervical epidural 10khz spinal cord stimulation on patients suffering from chronic, medically-refractory migraine

**DOI:** 10.1186/1129-2377-15-S1-G22

**Published:** 2014-09-18

**Authors:** S Palmisani, T Smith, R Arcioni, M Mercieri, V Vano, S Tigano, A Negro, A Al-Kaisy, P Martelletti

**Affiliations:** 1 Pain Management & Neuromodulation Centre, Guy’s & St Thomas’ NHS Trust, London, UK; 2 Department of Clinical and Molecular Medicine, Sapienza University of Rome, Rome, Italy

## 

A significant minority of chronic migraine (CM) patients do not respond to conventional medical treatment. Occipital nerve stimulation is a therapeutic option for refractory CM (rCM). However, randomized studies have failed to demonstrate efficacy. Cervical 10kHz spinal cord stimulation (10kHz-SCS) may provide a superior alternative to occipital stimulation. We report the preliminary results of a prospective, open-label, feasibility study to assess safety and tolerability cervical 10kHz-SCS in rCM patients.

The study had EC approval and the subjects gave informed consent. Included subjects were diagnosed with CM by an experienced headache specialist according to IHS guidelines, were refractory to medical treatments as defined by the Refractory Headache Special Interest Section of the AHS, and had failed Botox treatment. Medication Overuse headache was not excluded.

Patients underwent a 10kHz SCS-trial followed by a permanent implant if a significant reduction in headache intensity/episodes was reported during the trial. One or two epidural leads were used to cover the C2-C4.

At 6 months 7 out of 14 patients reported >30% reduction in headache days. The average headache days reduction from baseline was 6.9±7.3 days (p=0.04), while the reduction in the responder group was 12.9±5.3 days (p=0.001) . Three patients developed IPG tenderness and one had a lead migration that required surgical revision.

Paresthesia-free cervical 10kHz-SCS may be an effective therapeutic option for chronic migraineurs refractory to conventional medications and Botox treatment.

No conflict of interest.

